# Ethyl 1-(2-bromo­propano­yl)-4-hydr­oxy-2,6-diphenyl-1,2,5,6-tetra­hydro­pyridine-3-carboxylate

**DOI:** 10.1107/S1600536809023836

**Published:** 2009-06-27

**Authors:** G. Aridoss, D. Gayathri, D. Velmurugan, M. S. Kim, Yeon Tae Jeong

**Affiliations:** a Division of Image Science and Information Engineering, Pukyong National University, Busan 608-739, Republic of Korea; b Centre of Advanced Study in Crystallography and Biophysics, University of Madras, Guindy Campus, Chennai 600 025, India

## Abstract

The title compound, C_23_H_24_BrNO_4_, crystallizes with two independent mol­ecules per asymmetric unit. The methyl group of the ethoxy­carbonyl unit is disordered over two positions, with occupancies of 0.715 (12) and 0.285 (12) in one of the independent mol­ecules, and 0.529 (11) and 0.471 (11) in the other mol­ecule. In one of the independent mol­ecules, the tetra­hydro­pyridine ring adopts a half-chair conformation, while in the other it is in a distorted envelope conformation. In each independent mol­ecule, an intra­molecular O—H⋯O hydrogen bond generates an *S*(6) ring motif. The two independent mol­ecules are linked *via* C—H⋯O hydrogen bonds, forming a chain along the *c* axis.

## Related literature

For general background to the synthesis and properties of 2,6-diaryl­piperidin-4-one derivatives, see: Aridoss *et al.* (2007 ▶, 2008*b* ▶); Krishnakumar & Krishnapillay (1996 ▶); Krishnapillay *et al.* (2000 ▶); Rubiralta *et al.* (1991 ▶). For the biological activity of pyridine derivatives, see: Aridoss *et al.* (2008*a* ▶); Dewick (1997 ▶); Gwaltney *et al.* (2003 ▶); Michael (1997 ▶, 2001 ▶); Pinder (1992 ▶); Yeung *et al.* (1982 ▶). For a related structure, see: Subha Nandhini *et al.* (2003 ▶). For ring conformational analysis, see: Cremer & Pople (1975 ▶); Nardelli (1983 ▶).
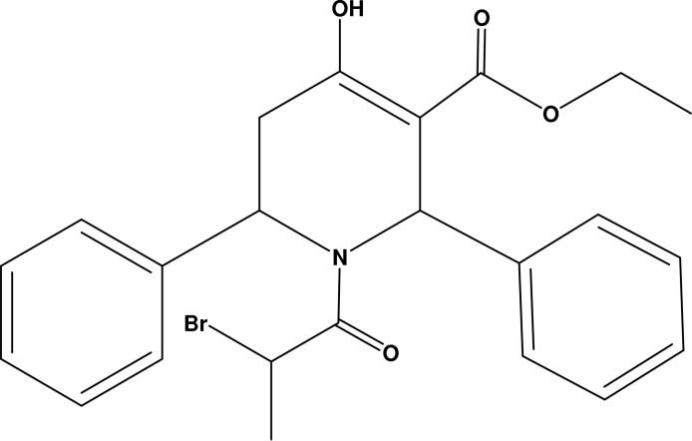


## Experimental

### 

#### Crystal data

C_23_H_24_BrNO_4_
*M* *_r_* = 458.34
Triclinic, 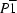

*a* = 10.3970 (4) Å
*b* = 14.4874 (6) Å
*c* = 15.8580 (7) Å
α = 65.457 (2)°
β = 89.556 (3)°
γ = 80.597 (3)°
*V* = 2138.80 (15) Å^3^
*Z* = 4
Mo *K*α radiation
μ = 1.95 mm^−1^
*T* = 293 K
0.30 × 0.16 × 0.16 mm

#### Data collection

Bruker Kappa APEXII area-detector diffractometer
Absorption correction: multi-scan (*SADABS*; Bruker, 1999 ▶) *T*_min_ = 0.600, *T*_max_ = 0.749
39210 measured reflections
7523 independent reflections
5075 reflections with *I* > 2σ(*I*)
*R*_int_ = 0.041

#### Refinement

*R*[*F* ^2^ > 2σ(*F* ^2^)] = 0.044
*wR*(*F* ^2^) = 0.138
*S* = 1.04
7523 reflections
545 parameters
28 restraints
H-atom parameters constrained
Δρ_max_ = 0.67 e Å^−3^
Δρ_min_ = −0.44 e Å^−3^

### 

Data collection: *APEX2* (Bruker, 2004 ▶); cell refinement: *SAINT* (Bruker, 2004 ▶); data reduction: *SAINT*; program(s) used to solve structure: *SIR92* (Altomare *et al.*, 1993 ▶); program(s) used to refine structure: *SHELXL97* (Sheldrick, 2008 ▶); molecular graphics: *PLATON* (Spek, 2009 ▶); software used to prepare material for publication: *SHELXL97*.

## Supplementary Material

Crystal structure: contains datablocks I, global. DOI: 10.1107/S1600536809023836/ci2830sup1.cif

Structure factors: contains datablocks I. DOI: 10.1107/S1600536809023836/ci2830Isup2.hkl

Additional supplementary materials:  crystallographic information; 3D view; checkCIF report

## Figures and Tables

**Table 1:** Hydrogen-bond geometry (Å, °)

*D*—H⋯*A*	*D*—H	H⋯*A*	*D*⋯*A*	*D*—H⋯*A*
O1—H1*O*⋯O3	0.82	1.83	2.547 (6)	146
O5—H5*O*⋯O7	0.82	1.90	2.582 (6)	140
C7—H7⋯O6^i^	0.93	2.58	3.349 (4)	141
C30—H30⋯O2^ii^	0.93	2.56	3.349 (5)	143

Symmetry codes: (i) 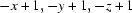; (ii) 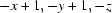.

**Fig. 1.**
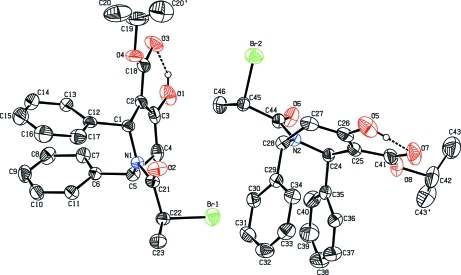
The molecular structure of title compound, showing 30% probability displacement ellipsoids. C-bound H atoms are not shown for clarity. All disorder components are shown.

**Fig. 2.**
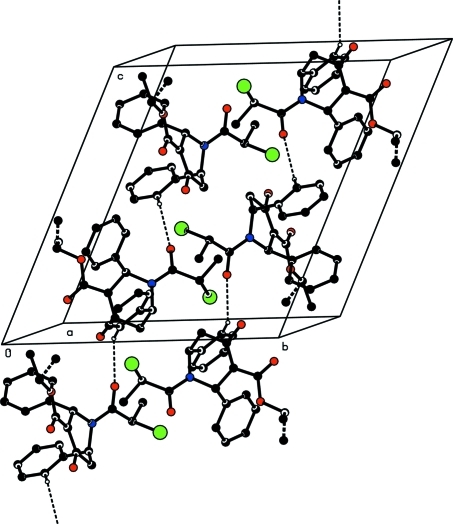
The crystal packing of the title compound. H atoms not involved in hydrogen bonding (dashed lines) have been omitted for clarity. All disorder components are shown.

## supplementary crystallographic information

### Comment

Hydroxy substituted nitrogen heterocycles containing six-membered rings are well known in nature and are components of many compounds with valuable pharmacological properties (Pinder, 1992; Michael, 1997). Functionalized tetrahydropyridines and piperidines are familiar substructures found in biologically active natural products and synthetic pharmaceuticals (Michael, 2001; Dewick, 1997; Pinder, 1992; Rubiralta *et al.*, 1991). *N*-Acyl and amino acyl derivatives of Δ^3^-tetrahydropyridine system and their derivatives were reported to act as farnesyltransferase inhibitors (Gwaltney *et al.*, 2003) and exhibit potent analgesic, anti-inflammatory and hyperglycemic effects (Yeung *et al.*, 1982) thereby attracted much attention in the pharmaceutical arena. Recently, we found that a *N*-chloroacetyl derivative of 3-carboxyethyl-2,6-diphenyl-4-hydroxy-Δ^3^-tetrahydropyridine was found to possess significant antibacterial activity against both Gram-positive and Gram-negative pathogens besides antitubercular activity (Aridoss *et al.*, 2008*a*). Unlike earlier reports on acylation of 2,6-diarylpiperidin-4-ones (Aridoss *et al.*, 2007, 2008*b*; Krishnakumar & Krishnapillay, 1996; Krishnapillay *et al.*, 2000), 2-bromopropionylation of 3-carboxyethyl-2,6- diphenylpiperidin-4-one gave tetrahydropyridine (title compound) through enolization across C3—C4 bond. In order to study the change in stereochemistry due to the introduction of double bond about C3—C4 besides 2-bromopropionylation, the tile compound was synthesized and X-ray crystal structure is discussed here.

The title compound crystallizes with two independent but closely similar molecules per asymmetric unit. The sums of angles around N1 (359.5 (8)°) and N2 (359.6 (8)°) are in accordance with *sp*^2^ hybridization. The C7—C6—C11, C13—C12—C17, C30—C29—C34 and C36—C35—C40 angles are slightly lower than the average value of 120°, as observed in Subha Nandhini *et al.* (2003). The N1/C1-C5 ring adopts a half-chair conformation while the N2/C24-C28 ring is in a distorted envelope conformation. The puckering parameters (Q, θ, φ; Cremer & Pople, 1975) and the smallest displacement asymmetry parameter (ΔC_2_(C2-C3); Nardelli, 1983) for the N1/C1-C5 ring are Q = 0.465 (4) Å, θ = 51.2 (5)°, φ = 317.9 (6)° and ΔC_2_(C2-C3) = 9.1 (5)°. The N2/C24-C28 ring adopts a distorted envelope conformation, with Q, θ, φ and ΔC_s_(C28) values of 0.478 (4)Å, 52.7 (5)°, 312.7 (6)° and 12.6 (4)°, respectively.

The molecular structure is stabilized by a strong O—H···O intramolecular interaction. In each independent molecule, the O—H···O hydrogen bond generates an S(6) motif. The crystal packing is stabilized by C—H···O intermolecular interactions. The two independent molecules are linked *via* C—H···O hydrogen bonds to form a chain along the *c* axis.

### Experimental

The title compound was obtained by adopting our earlier method (Aridoss *et al.*, 2007) with slight modification. To a cooled solution of 3-carboxyethyl-2,6-diphenylpiperidin-4-one (1 equiv.) and DMAP (1.5 equiv.) in dry dichloromethane, 2-bromopropionyl bromide (1 equiv.) in dry dichloromethane was added in drop wise under nitrogen atmosphere. Stirring was continued until the completion of reaction. Later, it was poured into water and extracted with dichloromethane. The combined organic extracts was then washed well with 3% sodium bicarbonate solution and dried over anhydrous sodium sulfate. This upon evaporation and purification by column chromatography gave two different isomers. The isomer with higher Rf value upon recrystallization in distilled ethanol afforded fine white crystals suitable for X-ray diffraction study. ^1^H NMR (400 MHz, CDCl_3_, p.p.m.): 12.44 (s, 1H, –OH); 7.16–6.82 (m, 11H, aromatic and H-2 protons); 5.31 (t, 1H, H-6); 4.48 (q, 1H, –CHBr); 4.12 (m, 2H, –CH_2_CH_3_); 2.94 (dd, 1H, H-5a); 2.84 (dd, 1H, H-5 e); 1.73 [3H, d, CH(Br)CH_3_]; 1.07 (t, 3H, CH_2_CH_3_).

### Refinement

The methyl group of the ethyl carboxylate unit is disordered over two positions in both independent molecules. The occupancies of major and minor components are 0.715 (12) and 0.285 (12) in molecule A, and 0.529 (11) and 0.471 (11) in molecule B. The C—C distances involving the disordered atoms were restrained to 1.53 (1) Å, and their displacement parameters were restrained to an approximate isotropic behaviour. All H-atoms were positioned geometrically and refined using a riding model, with O-H = 0.82 Å, C-H = 0.93-0.98 Å and *U*_iso_(H) = 1.5*U*_eq_(O,C_methyl_) and 1.2*U*_eq_(C) for other H atoms.

### Crystal data

**Table:**

C_23_H_24_BrNO_4_	*Z* = 4
*M**_r_* = 458.34	*F*(000) = 944
Triclinic, *P*1	*D*_x_ = 1.423 Mg m^−^^3^
Hall symbol: -P 1	Mo *K*α radiation, λ = 0.71073 Å
*a* = 10.3970 (4) Å	Cell parameters from 5388 reflections
*b* = 14.4874 (6) Å	θ = 1.4–25.0°
*c* = 15.8580 (7) Å	µ = 1.95 mm^−^^1^
α = 65.457 (2)°	*T* = 293 K
β = 89.556 (3)°	Prism, colourless
γ = 80.597 (3)°	0.30 × 0.16 × 0.16 mm
*V* = 2138.80 (15) Å^3^	

### Data collection

**Table:**

Bruker Kappa APEXII area-detector diffractometer	7523 independent reflections
Radiation source: fine-focus sealed tube	5075 reflections with *I* > 2σ(*I*)
graphite	*R*_int_ = 0.041
ω and φ scans	θ_max_ = 25.0°, θ_min_ = 1.4°
Absorption correction: multi-scan (*SADABS*; Bruker, 1999)	*h* = −12→12
*T*_min_ = 0.600, *T*_max_ = 0.749	*k* = −17→17
39210 measured reflections	*l* = −18→18

### Refinement

**Table:**

Refinement on *F*^2^	Primary atom site location: structure-invariant direct methods
Least-squares matrix: full	Secondary atom site location: difference Fourier map
*R*[*F*^2^ > 2σ(*F*^2^)] = 0.044	Hydrogen site location: inferred from neighbouring sites
*wR*(*F*^2^) = 0.138	H-atom parameters constrained
*S* = 1.04	*w* = 1/[σ^2^(*F*_o_^2^) + (0.0745*P*)^2^ + 0.8765*P*] where *P* = (*F*_o_^2^ + 2*F*_c_^2^)/3
7523 reflections	(Δ/σ)_max_ = 0.001
545 parameters	Δρ_max_ = 0.67 e Å^−^^3^
28 restraints	Δρ_min_ = −0.44 e Å^−^^3^

### Special details

**Table:**

Geometry. All e.s.d.'s (except the e.s.d. in the dihedral angle between two l.s. planes) are estimated using the full covariance matrix. The cell e.s.d.'s are taken into account individually in the estimation of e.s.d.'s in distances, angles and torsion angles; correlations between e.s.d.'s in cell parameters are only used when they are defined by crystal symmetry. An approximate (isotropic) treatment of cell e.s.d.'s is used for estimating e.s.d.'s involving l.s. planes.
Refinement. Refinement of *F*^2^ against ALL reflections. The weighted *R*-factor *wR* and goodness of fit *S* are based on *F*^2^, conventional *R*-factors *R* are based on *F*, with *F* set to zero for negative *F*^2^. The threshold expression of *F*^2^ > σ(*F*^2^) is used only for calculating *R*-factors(gt) *etc*. and is not relevant to the choice of reflections for refinement. *R*-factors based on *F*^2^ are statistically about twice as large as those based on *F*, and *R*- factors based on ALL data will be even larger.

### Fractional atomic coordinates and isotropic or equivalent isotropic displacement parameters (Å^2^)

**Table:**

	*x*	*y*	*z*	*U*_iso_*/*U*_eq_	Occ. (<1)
Br1	0.72815 (4)	0.34497 (3)	0.35318 (3)	0.06691 (16)	
O1	0.4992 (3)	0.6538 (3)	0.4898 (2)	0.0827 (9)	
H1O	0.4404	0.7031	0.4635	0.124*	
O2	0.7187 (3)	0.5800 (2)	0.18232 (17)	0.0648 (7)	
O3	0.3824 (3)	0.8162 (3)	0.3556 (3)	0.0932 (11)	
O4	0.4572 (2)	0.8545 (2)	0.2153 (3)	0.0735 (9)	
N1	0.7565 (2)	0.60133 (19)	0.31187 (18)	0.0376 (6)	
C1	0.6728 (3)	0.7045 (2)	0.2704 (2)	0.0410 (8)	
H1	0.6197	0.7058	0.2190	0.049*	
C2	0.5780 (3)	0.7178 (3)	0.3388 (3)	0.0471 (9)	
C3	0.5863 (4)	0.6480 (3)	0.4287 (3)	0.0551 (10)	
C4	0.6874 (4)	0.5545 (3)	0.4684 (3)	0.0537 (9)	
H4A	0.6504	0.4954	0.4730	0.064*	
H4B	0.7159	0.5431	0.5306	0.064*	
C5	0.8039 (3)	0.5633 (3)	0.4097 (2)	0.0413 (8)	
H5	0.8540	0.4936	0.4283	0.050*	
C6	0.8963 (3)	0.6295 (2)	0.4207 (2)	0.0380 (7)	
C7	0.8771 (4)	0.6770 (3)	0.4803 (2)	0.0495 (9)	
H7	0.8057	0.6688	0.5168	0.059*	
C8	0.9650 (4)	0.7371 (3)	0.4855 (3)	0.0637 (11)	
H8	0.9521	0.7689	0.5259	0.076*	
C9	1.0698 (4)	0.7505 (3)	0.4326 (3)	0.0657 (11)	
H9	1.1269	0.7923	0.4357	0.079*	
C10	1.0901 (4)	0.7022 (3)	0.3753 (3)	0.0620 (10)	
H10	1.1624	0.7101	0.3397	0.074*	
C11	1.0047 (3)	0.6414 (3)	0.3693 (3)	0.0500 (9)	
H11	1.0205	0.6082	0.3302	0.060*	
C12	0.7539 (3)	0.7893 (2)	0.2271 (2)	0.0436 (8)	
C13	0.7646 (3)	0.8605 (3)	0.2614 (3)	0.0545 (9)	
H13	0.7215	0.8575	0.3138	0.065*	
C14	0.8385 (4)	0.9366 (3)	0.2192 (4)	0.0774 (13)	
H14	0.8435	0.9848	0.2430	0.093*	
C15	0.9037 (5)	0.9417 (4)	0.1433 (4)	0.0887 (16)	
H15	0.9536	0.9929	0.1153	0.106*	
C16	0.8953 (5)	0.8708 (4)	0.1086 (3)	0.0835 (15)	
H16	0.9405	0.8733	0.0571	0.100*	
C17	0.8201 (4)	0.7953 (3)	0.1493 (3)	0.0640 (11)	
H17	0.8139	0.7482	0.1244	0.077*	
C18	0.4657 (4)	0.7992 (3)	0.3062 (4)	0.0636 (11)	
C19	0.3441 (5)	0.9369 (5)	0.1788 (5)	0.118 (2)	
H19A	0.3326	0.9762	0.2160	0.141*	0.715 (12)
H19B	0.2654	0.9089	0.1790	0.141*	0.715 (12)
H19C	0.3742	1.0012	0.1472	0.141*	0.285 (12)
H19D	0.2962	0.9402	0.2298	0.141*	0.285 (12)
C20	0.3715 (10)	1.0026 (7)	0.0831 (6)	0.129 (4)	0.715 (12)
H20A	0.2999	1.0589	0.0555	0.194*	0.715 (12)
H20B	0.3823	0.9626	0.0473	0.194*	0.715 (12)
H20C	0.4500	1.0290	0.0841	0.194*	0.715 (12)
C20'	0.261 (2)	0.924 (2)	0.1100 (16)	0.136 (11)	0.285 (12)
H20D	0.1882	0.9797	0.0870	0.204*	0.285 (12)
H20E	0.2296	0.8597	0.1393	0.204*	0.285 (12)
H20F	0.3117	0.9232	0.0593	0.204*	0.285 (12)
C21	0.7694 (3)	0.5460 (3)	0.2606 (2)	0.0429 (8)	
C22	0.8512 (3)	0.4390 (3)	0.3009 (3)	0.0474 (8)	
H22	0.9132	0.4323	0.3502	0.057*	
C23	0.9235 (4)	0.4149 (3)	0.2283 (3)	0.0686 (12)	
H23A	0.9844	0.4612	0.2030	0.103*	
H23B	0.8625	0.4228	0.1796	0.103*	
H23C	0.9701	0.3452	0.2559	0.103*	
Br2	0.16044 (4)	0.65873 (3)	0.14248 (3)	0.07332 (17)	
O5	0.0762 (3)	0.3339 (3)	0.0151 (2)	0.0923 (10)	
H5O	0.0614	0.2749	0.0371	0.139*	
O6	0.2515 (3)	0.4273 (2)	0.31396 (18)	0.0669 (8)	
O7	0.0454 (3)	0.1660 (3)	0.1541 (3)	0.0980 (11)	
O8	0.1301 (3)	0.1399 (2)	0.2932 (2)	0.0769 (9)	
N2	0.3001 (3)	0.40012 (19)	0.18645 (18)	0.0390 (6)	
C24	0.2625 (3)	0.2972 (2)	0.2320 (2)	0.0413 (8)	
H24	0.2060	0.2995	0.2811	0.050*	
C25	0.1797 (3)	0.2780 (3)	0.1653 (3)	0.0492 (9)	
C26	0.1563 (3)	0.3448 (3)	0.0751 (3)	0.0600 (11)	
C27	0.2110 (4)	0.4402 (3)	0.0324 (3)	0.0575 (10)	
H27A	0.1458	0.4982	0.0285	0.069*	
H27B	0.2332	0.4514	−0.0303	0.069*	
C28	0.3323 (3)	0.4348 (3)	0.0883 (2)	0.0435 (8)	
H28	0.3504	0.5050	0.0671	0.052*	
C29	0.4543 (3)	0.3678 (3)	0.0788 (2)	0.0425 (8)	
C30	0.4573 (4)	0.3175 (3)	0.0215 (3)	0.0553 (9)	
H30	0.3833	0.3265	−0.0156	0.066*	
C31	0.5702 (5)	0.2537 (3)	0.0191 (3)	0.0697 (12)	
H31	0.5712	0.2196	−0.0193	0.084*	
C32	0.6798 (4)	0.2401 (3)	0.0718 (3)	0.0730 (13)	
H32	0.7547	0.1957	0.0708	0.088*	
C33	0.6788 (4)	0.2924 (3)	0.1266 (3)	0.0662 (11)	
H33	0.7542	0.2850	0.1618	0.079*	
C34	0.5674 (3)	0.3556 (3)	0.1299 (3)	0.0541 (9)	
H34	0.5681	0.3908	0.1672	0.065*	
C35	0.3786 (3)	0.2134 (3)	0.2800 (2)	0.0465 (8)	
C36	0.4310 (4)	0.1441 (3)	0.2455 (3)	0.0595 (10)	
H36	0.3953	0.1492	0.1898	0.071*	
C37	0.5348 (5)	0.0677 (3)	0.2912 (4)	0.0865 (15)	
H37	0.5689	0.0213	0.2668	0.104*	
C38	0.5885 (5)	0.0596 (4)	0.3730 (5)	0.104 (2)	
H38	0.6590	0.0076	0.4044	0.125*	
C39	0.5380 (5)	0.1283 (4)	0.4084 (4)	0.0985 (17)	
H39	0.5745	0.1230	0.4640	0.118*	
C40	0.4342 (4)	0.2048 (3)	0.3626 (3)	0.0702 (12)	
H40	0.4007	0.2513	0.3871	0.084*	
C41	0.1131 (4)	0.1907 (3)	0.2014 (4)	0.0667 (12)	
C42	0.0768 (6)	0.0452 (4)	0.3394 (5)	0.1050 (19)	
H42A	0.1356	−0.0032	0.3919	0.126*	0.529 (11)
H42B	0.0697	0.0140	0.2965	0.126*	0.529 (11)
H42C	0.0936	0.0026	0.3063	0.126*	0.471 (11)
H42D	−0.0156	0.0599	0.3445	0.126*	0.471 (11)
C43	−0.0526 (9)	0.0668 (8)	0.3720 (9)	0.111 (4)	0.529 (11)
H43A	−0.1157	0.1007	0.3198	0.166*	0.529 (11)
H43B	−0.0495	0.1106	0.4034	0.166*	0.529 (11)
H43C	−0.0772	0.0033	0.4140	0.166*	0.529 (11)
C43'	0.1487 (14)	−0.0052 (9)	0.4351 (8)	0.120 (5)	0.471 (11)
H43D	0.1197	−0.0689	0.4713	0.180*	0.471 (11)
H43E	0.1310	0.0400	0.4656	0.180*	0.471 (11)
H43F	0.2410	−0.0186	0.4287	0.180*	0.471 (11)
C44	0.2887 (3)	0.4577 (3)	0.2355 (2)	0.0455 (8)	
C45	0.3253 (3)	0.5647 (3)	0.1922 (3)	0.0491 (9)	
H45	0.3835	0.5701	0.1421	0.059*	
C46	0.3890 (4)	0.5887 (3)	0.2638 (3)	0.0685 (12)	
H46A	0.4704	0.5422	0.2879	0.103*	
H46B	0.3324	0.5810	0.3135	0.103*	
H46C	0.4047	0.6583	0.2356	0.103*	

### Atomic displacement parameters (Å^2^)

**Table:**

	*U*^11^	*U*^22^	*U*^33^	*U*^12^	*U*^13^	*U*^23^
Br1	0.0709 (3)	0.0655 (3)	0.0582 (3)	−0.0183 (2)	0.0042 (2)	−0.0177 (2)
O1	0.068 (2)	0.121 (3)	0.088 (2)	−0.0393 (17)	0.0458 (17)	−0.064 (2)
O2	0.0806 (18)	0.0687 (17)	0.0456 (16)	0.0123 (14)	−0.0223 (14)	−0.0332 (14)
O3	0.0491 (16)	0.111 (3)	0.149 (3)	−0.0072 (16)	0.0303 (19)	−0.086 (3)
O4	0.0504 (16)	0.0612 (18)	0.104 (3)	0.0143 (13)	−0.0166 (16)	−0.0380 (18)
N1	0.0404 (14)	0.0404 (15)	0.0337 (15)	−0.0042 (11)	−0.0028 (11)	−0.0182 (12)
C1	0.0370 (17)	0.0418 (18)	0.046 (2)	−0.0028 (14)	−0.0027 (15)	−0.0213 (16)
C2	0.0354 (18)	0.053 (2)	0.065 (3)	−0.0104 (16)	0.0047 (16)	−0.035 (2)
C3	0.049 (2)	0.076 (3)	0.064 (3)	−0.033 (2)	0.0231 (19)	−0.045 (2)
C4	0.061 (2)	0.064 (2)	0.044 (2)	−0.030 (2)	0.0132 (17)	−0.0229 (19)
C5	0.053 (2)	0.0428 (18)	0.0294 (17)	−0.0081 (15)	−0.0015 (14)	−0.0166 (15)
C6	0.0404 (18)	0.0398 (18)	0.0307 (17)	−0.0057 (14)	−0.0036 (14)	−0.0123 (14)
C7	0.059 (2)	0.055 (2)	0.038 (2)	−0.0136 (17)	0.0025 (16)	−0.0230 (17)
C8	0.083 (3)	0.063 (3)	0.055 (3)	−0.015 (2)	−0.011 (2)	−0.034 (2)
C9	0.060 (3)	0.063 (3)	0.071 (3)	−0.024 (2)	−0.014 (2)	−0.020 (2)
C10	0.046 (2)	0.066 (3)	0.067 (3)	−0.0143 (19)	0.0022 (19)	−0.020 (2)
C11	0.046 (2)	0.056 (2)	0.049 (2)	−0.0073 (17)	0.0026 (17)	−0.0245 (18)
C12	0.0350 (17)	0.0401 (18)	0.045 (2)	0.0019 (14)	−0.0021 (15)	−0.0109 (16)
C13	0.046 (2)	0.045 (2)	0.072 (3)	−0.0071 (16)	−0.0014 (18)	−0.024 (2)
C14	0.069 (3)	0.053 (3)	0.106 (4)	−0.016 (2)	−0.001 (3)	−0.028 (3)
C15	0.072 (3)	0.059 (3)	0.105 (4)	−0.019 (2)	0.011 (3)	−0.002 (3)
C16	0.079 (3)	0.073 (3)	0.068 (3)	−0.009 (3)	0.027 (2)	−0.002 (3)
C17	0.067 (3)	0.058 (2)	0.055 (3)	−0.005 (2)	0.007 (2)	−0.014 (2)
C18	0.042 (2)	0.065 (3)	0.102 (4)	−0.0127 (19)	0.004 (2)	−0.052 (3)
C19	0.079 (4)	0.093 (4)	0.176 (7)	0.033 (3)	−0.037 (4)	−0.069 (5)
C20	0.141 (7)	0.106 (6)	0.108 (7)	0.044 (5)	−0.038 (5)	−0.035 (5)
C20'	0.127 (13)	0.137 (13)	0.139 (13)	−0.004 (9)	−0.002 (9)	−0.058 (9)
C21	0.0409 (18)	0.051 (2)	0.041 (2)	−0.0022 (15)	−0.0047 (15)	−0.0254 (17)
C22	0.0458 (19)	0.050 (2)	0.054 (2)	−0.0019 (16)	−0.0067 (16)	−0.0311 (18)
C23	0.063 (2)	0.066 (3)	0.086 (3)	−0.006 (2)	0.018 (2)	−0.043 (2)
Br2	0.0654 (3)	0.0715 (3)	0.0637 (3)	−0.0013 (2)	0.0025 (2)	−0.0133 (2)
O5	0.0693 (19)	0.146 (3)	0.086 (2)	−0.019 (2)	−0.0188 (18)	−0.071 (2)
O6	0.107 (2)	0.0668 (17)	0.0462 (16)	−0.0365 (16)	0.0308 (15)	−0.0355 (14)
O7	0.084 (2)	0.125 (3)	0.130 (3)	−0.052 (2)	0.011 (2)	−0.086 (3)
O8	0.080 (2)	0.074 (2)	0.091 (2)	−0.0448 (16)	0.0195 (17)	−0.0374 (18)
N2	0.0472 (15)	0.0416 (15)	0.0321 (15)	−0.0098 (12)	0.0087 (12)	−0.0187 (12)
C24	0.0440 (18)	0.0433 (18)	0.045 (2)	−0.0132 (15)	0.0107 (15)	−0.0243 (16)
C25	0.0410 (19)	0.061 (2)	0.059 (2)	−0.0073 (17)	0.0024 (17)	−0.038 (2)
C26	0.042 (2)	0.086 (3)	0.068 (3)	0.000 (2)	−0.0036 (19)	−0.053 (3)
C27	0.054 (2)	0.074 (3)	0.039 (2)	0.006 (2)	−0.0034 (17)	−0.024 (2)
C28	0.055 (2)	0.0434 (19)	0.0313 (18)	−0.0079 (15)	0.0073 (15)	−0.0148 (15)
C29	0.0487 (19)	0.048 (2)	0.0326 (18)	−0.0119 (15)	0.0126 (15)	−0.0178 (16)
C30	0.061 (2)	0.067 (2)	0.042 (2)	−0.0076 (19)	0.0085 (17)	−0.0279 (19)
C31	0.086 (3)	0.071 (3)	0.063 (3)	−0.013 (2)	0.029 (2)	−0.040 (2)
C32	0.064 (3)	0.070 (3)	0.071 (3)	0.000 (2)	0.026 (2)	−0.021 (2)
C33	0.049 (2)	0.073 (3)	0.067 (3)	−0.008 (2)	0.005 (2)	−0.022 (2)
C34	0.052 (2)	0.059 (2)	0.057 (2)	−0.0147 (18)	0.0058 (18)	−0.0273 (19)
C35	0.0490 (19)	0.0395 (19)	0.049 (2)	−0.0156 (15)	0.0076 (16)	−0.0140 (17)
C36	0.059 (2)	0.048 (2)	0.069 (3)	−0.0082 (18)	0.008 (2)	−0.023 (2)
C37	0.073 (3)	0.058 (3)	0.111 (4)	0.004 (2)	0.006 (3)	−0.023 (3)
C38	0.071 (3)	0.065 (3)	0.131 (6)	0.008 (3)	−0.017 (3)	−0.004 (3)
C39	0.091 (4)	0.084 (4)	0.091 (4)	−0.007 (3)	−0.035 (3)	−0.011 (3)
C40	0.077 (3)	0.063 (3)	0.067 (3)	−0.009 (2)	−0.010 (2)	−0.025 (2)
C41	0.048 (2)	0.076 (3)	0.100 (4)	−0.017 (2)	0.010 (2)	−0.058 (3)
C42	0.108 (4)	0.078 (3)	0.142 (6)	−0.052 (3)	0.026 (4)	−0.046 (4)
C43	0.116 (7)	0.096 (7)	0.122 (8)	−0.039 (5)	0.028 (6)	−0.040 (6)
C43'	0.151 (9)	0.087 (7)	0.123 (9)	−0.039 (6)	0.007 (7)	−0.038 (6)
C44	0.050 (2)	0.050 (2)	0.044 (2)	−0.0150 (16)	0.0105 (16)	−0.0256 (17)
C45	0.050 (2)	0.048 (2)	0.059 (2)	−0.0145 (16)	0.0174 (17)	−0.0295 (18)
C46	0.065 (3)	0.069 (3)	0.087 (3)	−0.023 (2)	−0.001 (2)	−0.043 (2)

### Geometric parameters (Å, °)

**Table:**

Br1—C22	1.948 (4)	Br2—C45	1.945 (4)
O1—C3	1.340 (4)	O5—C26	1.343 (4)
O1—H1O	0.82	O5—H5O	0.82
O2—C21	1.216 (4)	O6—C44	1.218 (4)
O3—C18	1.227 (5)	O7—C41	1.223 (5)
O4—C18	1.322 (5)	O8—C41	1.329 (6)
O4—C19	1.456 (5)	O8—C42	1.460 (5)
N1—C21	1.352 (4)	N2—C44	1.350 (4)
N1—C5	1.472 (4)	N2—C28	1.478 (4)
N1—C1	1.480 (4)	N2—C24	1.480 (4)
C1—C2	1.511 (5)	C24—C25	1.508 (5)
C1—C12	1.524 (5)	C24—C35	1.509 (5)
C1—H1	0.98	C24—H24	0.98
C2—C3	1.356 (5)	C25—C26	1.348 (6)
C2—C18	1.440 (5)	C25—C41	1.445 (5)
C3—C4	1.474 (6)	C26—C27	1.472 (6)
C4—C5	1.511 (5)	C27—C28	1.519 (5)
C4—H4A	0.97	C27—H27A	0.97
C4—H4B	0.97	C27—H27B	0.97
C5—C6	1.521 (4)	C28—C29	1.513 (5)
C5—H5	0.98	C28—H28	0.98
C6—C11	1.377 (5)	C29—C34	1.377 (5)
C6—C7	1.378 (5)	C29—C30	1.379 (5)
C7—C8	1.387 (5)	C30—C31	1.381 (6)
C7—H7	0.93	C30—H30	0.93
C8—C9	1.359 (6)	C31—C32	1.357 (6)
C8—H8	0.93	C31—H31	0.93
C9—C10	1.355 (6)	C32—C33	1.369 (6)
C9—H9	0.93	C32—H32	0.93
C10—C11	1.377 (5)	C33—C34	1.369 (5)
C10—H10	0.93	C33—H33	0.93
C11—H11	0.93	C34—H34	0.93
C12—C13	1.370 (5)	C35—C36	1.371 (5)
C12—C17	1.385 (5)	C35—C40	1.384 (5)
C13—C14	1.382 (6)	C36—C37	1.368 (6)
C13—H13	0.93	C36—H36	0.93
C14—C15	1.358 (7)	C37—C38	1.367 (8)
C14—H14	0.93	C37—H37	0.93
C15—C16	1.367 (7)	C38—C39	1.368 (8)
C15—H15	0.93	C38—H38	0.93
C16—C17	1.381 (6)	C39—C40	1.369 (6)
C16—H16	0.93	C39—H39	0.93
C17—H17	0.93	C40—H40	0.93
C19—C20	1.475 (8)	C42—C43	1.470 (8)
C19—C20'	1.488 (10)	C42—C43'	1.521 (9)
C19—H19A	0.97	C42—H42A	0.97
C19—H19B	0.97	C42—H42B	0.97
C19—H19C	0.96	C42—H42C	0.96
C19—H19D	0.96	C42—H42D	0.96
C20—H20A	0.96	C43—H43A	0.96
C20—H20B	0.96	C43—H43B	0.96
C20—H20C	0.96	C43—H43C	0.96
C20'—H20D	0.96	C43'—H43D	0.96
C20'—H20E	0.96	C43'—H43E	0.96
C20'—H20F	0.96	C43'—H43F	0.96
C21—C22	1.512 (5)	C44—C45	1.523 (5)
C22—C23	1.500 (5)	C45—C46	1.505 (5)
C22—H22	0.98	C45—H45	0.98
C23—H23A	0.96	C46—H46A	0.96
C23—H23B	0.96	C46—H46B	0.96
C23—H23C	0.96	C46—H46C	0.96
			
C3—O1—H1O	109.5	C26—O5—H5O	109.5
C18—O4—C19	115.5 (4)	C41—O8—C42	119.2 (4)
C21—N1—C5	125.7 (3)	C44—N2—C28	125.9 (3)
C21—N1—C1	117.2 (3)	C44—N2—C24	116.6 (3)
C5—N1—C1	116.6 (2)	C28—N2—C24	117.1 (2)
N1—C1—C2	110.5 (3)	N2—C24—C25	110.5 (3)
N1—C1—C12	111.4 (2)	N2—C24—C35	112.1 (2)
C2—C1—C12	115.7 (3)	C25—C24—C35	115.0 (3)
N1—C1—H1	106.2	N2—C24—H24	106.2
C2—C1—H1	106.2	C25—C24—H24	106.2
C12—C1—H1	106.2	C35—C24—H24	106.2
C3—C2—C18	118.1 (4)	C26—C25—C41	119.0 (4)
C3—C2—C1	121.9 (3)	C26—C25—C24	122.1 (3)
C18—C2—C1	119.5 (4)	C41—C25—C24	118.6 (3)
O1—C3—C2	123.5 (4)	O5—C26—C25	123.8 (4)
O1—C3—C4	112.7 (4)	O5—C26—C27	112.3 (4)
C2—C3—C4	123.7 (3)	C25—C26—C27	123.8 (3)
C3—C4—C5	111.7 (3)	C26—C27—C28	111.3 (3)
C3—C4—H4A	109.3	C26—C27—H27A	109.4
C5—C4—H4A	109.3	C28—C27—H27A	109.4
C3—C4—H4B	109.3	C26—C27—H27B	109.4
C5—C4—H4B	109.3	C28—C27—H27B	109.4
H4A—C4—H4B	107.9	H27A—C27—H27B	108.0
N1—C5—C4	108.6 (3)	N2—C28—C29	111.1 (3)
N1—C5—C6	110.8 (3)	N2—C28—C27	107.5 (3)
C4—C5—C6	114.9 (3)	C29—C28—C27	115.3 (3)
N1—C5—H5	107.4	N2—C28—H28	107.6
C4—C5—H5	107.4	C29—C28—H28	107.6
C6—C5—H5	107.4	C27—C28—H28	107.6
C11—C6—C7	118.6 (3)	C34—C29—C30	118.2 (3)
C11—C6—C5	118.3 (3)	C34—C29—C28	119.0 (3)
C7—C6—C5	123.0 (3)	C30—C29—C28	122.8 (3)
C6—C7—C8	119.5 (4)	C29—C30—C31	120.0 (4)
C6—C7—H7	120.2	C29—C30—H30	120.0
C8—C7—H7	120.2	C31—C30—H30	120.0
C9—C8—C7	121.2 (4)	C32—C31—C30	121.1 (4)
C9—C8—H8	119.4	C32—C31—H31	119.5
C7—C8—H8	119.4	C30—C31—H31	119.5
C10—C9—C8	119.3 (4)	C31—C32—C33	119.3 (4)
C10—C9—H9	120.4	C31—C32—H32	120.4
C8—C9—H9	120.4	C33—C32—H32	120.4
C9—C10—C11	120.7 (4)	C32—C33—C34	120.2 (4)
C9—C10—H10	119.7	C32—C33—H33	119.9
C11—C10—H10	119.7	C34—C33—H33	119.9
C6—C11—C10	120.6 (4)	C33—C34—C29	121.2 (4)
C6—C11—H11	119.7	C33—C34—H34	119.4
C10—C11—H11	119.7	C29—C34—H34	119.4
C13—C12—C17	118.1 (3)	C36—C35—C40	118.2 (4)
C13—C12—C1	123.0 (3)	C36—C35—C24	122.4 (3)
C17—C12—C1	118.8 (3)	C40—C35—C24	119.3 (3)
C12—C13—C14	121.0 (4)	C37—C36—C35	121.4 (5)
C12—C13—H13	119.5	C37—C36—H36	119.3
C14—C13—H13	119.5	C35—C36—H36	119.3
C15—C14—C13	120.6 (5)	C38—C37—C36	119.9 (5)
C15—C14—H14	119.7	C38—C37—H37	120.0
C13—C14—H14	119.7	C36—C37—H37	120.0
C14—C15—C16	119.3 (4)	C37—C38—C39	119.7 (5)
C14—C15—H15	120.4	C37—C38—H38	120.2
C16—C15—H15	120.4	C39—C38—H38	120.2
C15—C16—C17	120.6 (5)	C38—C39—C40	120.4 (5)
C15—C16—H16	119.7	C38—C39—H39	119.8
C17—C16—H16	119.7	C40—C39—H39	119.8
C16—C17—C12	120.4 (4)	C39—C40—C35	120.5 (5)
C16—C17—H17	119.8	C39—C40—H40	119.8
C12—C17—H17	119.8	C35—C40—H40	119.8
O3—C18—O4	121.6 (4)	O7—C41—O8	122.6 (4)
O3—C18—C2	124.8 (5)	O7—C41—C25	124.7 (5)
O4—C18—C2	113.5 (4)	O8—C41—C25	112.7 (3)
O4—C19—C20	105.7 (6)	O8—C42—C43	110.7 (6)
O4—C19—C20'	111.8 (10)	O8—C42—C43'	103.2 (6)
C20—C19—C20'	69.1 (12)	C43—C42—C43'	95.8 (9)
O4—C19—H19A	110.6	O8—C42—H42A	109.5
C20—C19—H19A	110.6	C43—C42—H42A	109.5
C20'—C19—H19A	135.7	O8—C42—H42B	109.5
O4—C19—H19B	110.6	C43—C42—H42B	109.5
C20—C19—H19B	110.6	C43'—C42—H42B	127.0
H19A—C19—H19B	108.7	H42A—C42—H42B	108.1
O4—C19—H19C	108.6	O8—C42—H42C	110.7
C20'—C19—H19C	107.3	C43—C42—H42C	122.1
H19A—C19—H19C	69.9	C43'—C42—H42C	111.8
H19B—C19—H19C	138.0	H42A—C42—H42C	92.5
O4—C19—H19D	108.9	O8—C42—H42D	111.3
C20—C19—H19D	141.1	C43'—C42—H42D	110.6
C20'—C19—H19D	112.2	H42A—C42—H42D	122.1
H19B—C19—H19D	72.8	H42B—C42—H42D	95.0
H19C—C19—H19D	107.8	H42C—C42—H42D	109.1
C19—C20—H20A	109.5	C42—C43—H43A	109.5
H19C—C20—H20A	98.0	H42D—C43—H43A	85.4
C19—C20—H20B	109.5	C42—C43—H43B	109.5
H19C—C20—H20B	146.1	H42D—C43—H43B	128.5
H20A—C20—H20B	109.5	H43A—C43—H43B	109.5
C19—C20—H20C	109.5	C42—C43—H43C	109.5
H19C—C20—H20C	78.1	H42D—C43—H43C	111.0
H20A—C20—H20C	109.5	H43A—C43—H43C	109.5
H20B—C20—H20C	109.5	H43B—C43—H43C	109.5
C19—C20'—H20D	109.5	C42—C43'—H43D	109.5
C19—C20'—H20E	109.5	C42—C43'—H43E	109.5
H20D—C20'—H20E	109.5	H43D—C43'—H43E	109.5
C19—C20'—H20F	109.5	C42—C43'—H43F	109.5
H20D—C20'—H20F	109.5	H43D—C43'—H43F	109.5
H20E—C20'—H20F	109.5	H43E—C43'—H43F	109.5
O2—C21—N1	122.1 (3)	O6—C44—N2	122.6 (3)
O2—C21—C22	118.7 (3)	O6—C44—C45	118.2 (3)
N1—C21—C22	119.2 (3)	N2—C44—C45	119.1 (3)
C23—C22—C21	112.0 (3)	C46—C45—C44	111.3 (3)
C23—C22—Br1	109.7 (2)	C46—C45—Br2	109.9 (2)
C21—C22—Br1	105.6 (2)	C44—C45—Br2	104.9 (2)
C23—C22—H22	109.8	C46—C45—H45	110.2
C21—C22—H22	109.8	C44—C45—H45	110.2
Br1—C22—H22	109.8	Br2—C45—H45	110.2
C22—C23—H23A	109.5	C45—C46—H46A	109.5
C22—C23—H23B	109.5	C45—C46—H46B	109.5
H23A—C23—H23B	109.5	H46A—C46—H46B	109.5
C22—C23—H23C	109.5	C45—C46—H46C	109.5
H23A—C23—H23C	109.5	H46A—C46—H46C	109.5
H23B—C23—H23C	109.5	H46B—C46—H46C	109.5
			
C21—N1—C1—C2	131.6 (3)	C44—N2—C24—C25	135.0 (3)
C5—N1—C1—C2	−40.7 (3)	C28—N2—C24—C25	−38.4 (4)
C21—N1—C1—C12	−98.3 (3)	C44—N2—C24—C35	−95.2 (3)
C5—N1—C1—C12	89.4 (3)	C28—N2—C24—C35	91.4 (3)
N1—C1—C2—C3	8.5 (4)	N2—C24—C25—C26	5.3 (4)
C12—C1—C2—C3	−119.3 (3)	C35—C24—C25—C26	−122.9 (4)
N1—C1—C2—C18	−163.3 (3)	N2—C24—C25—C41	−168.4 (3)
C12—C1—C2—C18	68.9 (4)	C35—C24—C25—C41	63.4 (4)
C18—C2—C3—O1	−3.7 (5)	C41—C25—C26—O5	−1.6 (6)
C1—C2—C3—O1	−175.6 (3)	C24—C25—C26—O5	−175.4 (3)
C18—C2—C3—C4	172.7 (3)	C41—C25—C26—C27	175.4 (3)
C1—C2—C3—C4	0.8 (5)	C24—C25—C26—C27	1.7 (5)
O1—C3—C4—C5	−163.7 (3)	O5—C26—C27—C28	−161.1 (3)
C2—C3—C4—C5	19.5 (5)	C25—C26—C27—C28	21.6 (5)
C21—N1—C5—C4	−110.1 (3)	C44—N2—C28—C29	121.8 (3)
C1—N1—C5—C4	61.6 (3)	C24—N2—C28—C29	−65.5 (4)
C21—N1—C5—C6	122.8 (3)	C44—N2—C28—C27	−111.3 (4)
C1—N1—C5—C6	−65.5 (3)	C24—N2—C28—C27	61.4 (4)
C3—C4—C5—N1	−47.5 (4)	C26—C27—C28—N2	−49.5 (4)
C3—C4—C5—C6	77.2 (4)	C26—C27—C28—C29	74.9 (4)
N1—C5—C6—C11	−56.3 (4)	N2—C28—C29—C34	−55.0 (4)
C4—C5—C6—C11	−179.9 (3)	C27—C28—C29—C34	−177.6 (3)
N1—C5—C6—C7	124.4 (3)	N2—C28—C29—C30	124.5 (3)
C4—C5—C6—C7	0.8 (5)	C27—C28—C29—C30	1.9 (5)
C11—C6—C7—C8	1.5 (5)	C34—C29—C30—C31	2.5 (5)
C5—C6—C7—C8	−179.2 (3)	C28—C29—C30—C31	−177.0 (3)
C6—C7—C8—C9	0.2 (6)	C29—C30—C31—C32	−0.6 (6)
C7—C8—C9—C10	−1.5 (6)	C30—C31—C32—C33	−1.5 (7)
C8—C9—C10—C11	1.0 (6)	C31—C32—C33—C34	1.7 (6)
C7—C6—C11—C10	−2.0 (5)	C32—C33—C34—C29	0.2 (6)
C5—C6—C11—C10	178.7 (3)	C30—C29—C34—C33	−2.3 (5)
C9—C10—C11—C6	0.7 (6)	C28—C29—C34—C33	177.2 (3)
N1—C1—C12—C13	−112.9 (3)	N2—C24—C35—C36	−106.6 (4)
C2—C1—C12—C13	14.4 (4)	C25—C24—C35—C36	20.8 (5)
N1—C1—C12—C17	67.2 (4)	N2—C24—C35—C40	74.1 (4)
C2—C1—C12—C17	−165.5 (3)	C25—C24—C35—C40	−158.5 (3)
C17—C12—C13—C14	0.5 (5)	C40—C35—C36—C37	0.5 (6)
C1—C12—C13—C14	−179.4 (3)	C24—C35—C36—C37	−178.8 (4)
C12—C13—C14—C15	−0.9 (6)	C35—C36—C37—C38	−0.2 (7)
C13—C14—C15—C16	0.3 (7)	C36—C37—C38—C39	−0.1 (8)
C14—C15—C16—C17	0.7 (7)	C37—C38—C39—C40	0.1 (9)
C15—C16—C17—C12	−1.1 (7)	C38—C39—C40—C35	0.2 (8)
C13—C12—C17—C16	0.5 (5)	C36—C35—C40—C39	−0.5 (6)
C1—C12—C17—C16	−179.6 (3)	C24—C35—C40—C39	178.8 (4)
C19—O4—C18—O3	0.8 (6)	C42—O8—C41—O7	6.0 (6)
C19—O4—C18—C2	179.2 (4)	C42—O8—C41—C25	−175.5 (4)
C3—C2—C18—O3	7.2 (6)	C26—C25—C41—O7	7.6 (6)
C1—C2—C18—O3	179.3 (3)	C24—C25—C41—O7	−178.5 (4)
C3—C2—C18—O4	−171.1 (3)	C26—C25—C41—O8	−170.9 (3)
C1—C2—C18—O4	1.0 (5)	C24—C25—C41—O8	3.1 (5)
C18—O4—C19—C20	166.3 (6)	C41—O8—C42—C43	−94.8 (8)
C18—O4—C19—C20'	−120.4 (13)	C41—O8—C42—C43'	163.7 (7)
C5—N1—C21—O2	174.0 (3)	C28—N2—C44—O6	173.9 (3)
C1—N1—C21—O2	2.4 (5)	C24—N2—C44—O6	1.1 (5)
C5—N1—C21—C22	−6.9 (5)	C28—N2—C44—C45	−7.0 (5)
C1—N1—C21—C22	−178.5 (3)	C24—N2—C44—C45	−179.7 (3)
O2—C21—C22—C23	34.5 (5)	O6—C44—C45—C46	35.6 (5)
N1—C21—C22—C23	−144.6 (3)	N2—C44—C45—C46	−143.6 (3)
O2—C21—C22—Br1	−84.9 (3)	O6—C44—C45—Br2	−83.2 (4)
N1—C21—C22—Br1	96.0 (3)	N2—C44—C45—Br2	97.7 (3)

### Hydrogen-bond geometry (Å, °)

**Table:**

*D*—H···*A*	*D*—H	H···*A*	*D*···*A*	*D*—H···*A*
O1—H1O···O3	0.82	1.83	2.547 (6)	146
O5—H5O···O7	0.82	1.90	2.582 (6)	140
C7—H7···O6^i^	0.93	2.58	3.349 (4)	141
C30—H30···O2^ii^	0.93	2.56	3.349 (5)	143

Symmetry codes: (i) −*x*+1, −*y*+1, −*z*+1; (ii) −*x*+1, −*y*+1, −*z*.
